# Quantitative Analysis of Cold Stress Inducing Lipidomic Changes in *Shewanella putrefaciens* Using UHPLC-ESI-MS/MS

**DOI:** 10.3390/molecules24244609

**Published:** 2019-12-16

**Authors:** Xin Gao, Wenru Liu, Jun Mei, Jing Xie

**Affiliations:** 1 College of Food Science and Technology, Shanghai Ocean University, Shanghai 201306, China; gxdg@163.com (X.G.); wenrul@163.com (W.L.); 2 National Experimental Teaching Demonstration Center for Food Science Engineering, Shanghai Ocean University, Shanghai 201306, China; 3 Shanghai Engineering Research Center of Aquatic Product Processing and Preservation, Shanghai 201306, China; 4 Shanghai Professional Technology Service Platform on Cold Chain Equipment Performance and Energy Saving Evaluation, Shanghai 201306, China; 5 School of Health and Social Care, Shanghai Urban Construction Vocational College, Shanghai 201415, China

**Keywords:** *Shewanella putrefaciens*, lipidomic analysis, cold adaption tolerance, phospholipids

## Abstract

*Shewanella putrefaciens* is a well-known specific spoilage organism (SSO) and cold-tolerant microorganism in refrigerated fresh marine fish. Cold-adapted mechanism includes increased fluidity of lipid membranes by the ability to finely adjust lipids composition. In the present study, the lipid profile of *S. putrefaciens* cultivated at 30, 20, 10, 4, and 0 °C was explored using ultra-high-pressure liquid chromatography/electrospray ionization tandem mass spectrometry (UHPLC-ESI-MS/MS) to discuss the effect of lipid composition on cold-adapted tolerance. Lipidomic analysis detected a total of 27 lipid classes and 606 lipid molecular species in *S. putrefaciens* cultivated at 30, 20, 10, 4, and 0 °C. *S. putrefaciens* cultivated at 30 °C (SP-30) had significantly higher content of glycerolipids, sphingolipids, saccharolipids, and fatty acids compared with that at 0 °C (SP-0); however, the lower content of phospholipids (13.97%) was also found in SP-30. PE (30:0), PE (15:0/15:0), PE (31:0), PA (33:1), PE (32:1), PE (33:1), PE (25:0), PC (22:0), PE (29:0), PE (34:1), dMePE (15:0/16:1), PE (31:1), dMePE (15:1/15:0), PG (34:2), and PC (11:0/11:0) were identified as the most abundant lipid molecular species in *S. putrefaciens* cultivated at 30, 20, 10, 4, and 0 °C. The increase of PG content contributes to the construction of membrane lipid bilayer and successfully maintains membrane integrity under cold stress. *S. putrefaciens* cultivated at low temperature significantly increased the total unsaturated liquid contents but decreased the content of saturated liquid contents.

## 1. Introduction

Bacterial cell membranes are mainly composed of glycerolipids such as phospholipids (PL) and glycolipids (GL), which play an important role in membrane properties and functions [1]. Membrane lipid homeostasis and adaptation to changing environmental conditions (including temperature, oxygen, pressure, and so on) are essential for bacterial survival [2,3]. However, some researchers mainly focused on the fatty acids composition of the bacterial cell membrane instead of the lipids [4,5]. Most of the fatty acids in bacteria could be esterified to lipids, such as PL and GL, which have been largely ignored [6]. The terms “lipidome” and “lipidomics” were firstly introduced by Kishimoto et al. [7] and then defined from Han and Gross [8]. According to these authors, the aim of lipidomics is “the full characterization of lipid molecular species and of their biological roles with respect to expression of proteins involved in lipid metabolism and function” [9]. However, only a few studies reported the lipidomics of bacteria under cold stress, which are possibly due to the complexity and distinct types of lipids that can be found in different bacteria. Also, the most commonly used methods for the analysis of lipids in bacteria are thin-layer chromatography (TLC, reviewed by Fuchs [10]), gas chromatography (GC) [11,12], and nuclear magnetic resonance (NMR) [13,14], which offer limited information. Now, some methods based on mass spectrometry (MS) have been used for the detailed analysis of bacterial cell membranes lipidomics, including directly analyzing lipid extracts by matrix-assisted laser desorption ionization (MALDI) [15,16], liquid chromatography (LC) [17], and electrospray ionization (ESI) [16,18,19] coupled to MS. The introduction of UHPLC coupled to tandem MS (UHPLC-MS/MS) allows rapid and effective separation of individual lipid species and has been become a powerful tool for analyzing lipid classes in bacteria [20].

*Shewanella putrefaciens* is a Gram-negative, rod-shaped bacterium and a well-known specific spoilage organism (SSO) of refrigerated fresh marine fish [21], such as *Pseudosciaena crocea* [22,23], *Paralichthys olivaceus* [24], *Oncorhynchus kisutch* [25], *Sparus aurata* [26], *Thunnus albacares, Salmo salar* [27], *Rachycentron canadum* [28], and so on. *S. putrefaciens* could grow on fish during cold storage and produce large amounts of trimethylamine (TMA) with the characteristic “fishy” aroma [29,30]. Furthermore, quality degradation in marine fish muscle may also lead to other amine compounds (ammonia, methylamine (MA), and dimethylamine (DMA), etc.) which all induce an “off-flavor” in marine fish [31,32]. This “fishy” aroma could generalize associations with fish spoilage and have significant adverse effects on the marine fish consumption. *S. putrefaciens* is a cold-adapted microorganism in refrigerated marine fish, and cold-adapted microorganisms exhibit many unique characteristics and molecular mechanisms that allow them to adapt to the environment [33,34]. Low temperature presents many challenges for cold-adapted microorganisms to grow at low temperature, including the increased liquid water viscosity, decreased enzyme activity, reduced lipid membranes’ fluidity, enhanced the stability of inhibiting nucleic acid structure, and disturbed protein conformation [35,36,37,38].

However, until now, no studies have addressed cold adaptation in *S. putrefaciens*. The aim of the present study is to analyze the changes in the content, composition, and saturation levels of lipids in *S. putrefaciens* cultivated at 30, 20, 10, 4, and 0 °C using lipidomic method and to identify the major lipids and molecular species that are induced or enriched due to cold stress.

## 2. Materials and Methods

### 2.1. Pretreatment of Samples

Broth cultures of *S. putrefaciens* (ATCC 8071) were prepared as follows: 1 mL aliquots of logarithmic phase grown broth cultures were transferred to 250 mL erlenmeyer flasks containing 100 mL medium. The flasks were incubated aerobically agitating at 200 rpm, at 30, 20, 10, 4, and 0 °C, until an absorbance (OD600) of 0.4 was attained. The bacterial cells were then harvested by centrifugation (11,960× *g*, 20 min), rinsed in phosphate buffer saline (pH 7.0) and stored at −80 °C until use.

Cells of *S. putrefaciens* ATCC 8071 were resuspended in 400 μL ice-cold 75% methanol solution and sonicated for 15 min at 200 W using a high intensity probe sonicator (UP-250S sonicator, Scientz, Ningbo, China). Then, the mixture was fully vortex oscillated with 1 mL ice-cold methyl tert-butyl ether (MTBE) and rotated at 4 °C for 1 h. After sonicating for 15 min, 250 μL of ultrapure water was added and oscillated for 1 min and incubated at room temperature for 10 min. Mixtures were centrifuged at 14,000× *g* at 4 °C for 15 min. Lipids in the organic phase were separated and evaporated by nitrogen flow. The separated lipids extract were re-dissolved in isopropanol/methanol (1:1, *v*/*v*) solutions. All the samples were repeated six times.

### 2.2. Lipids Separation by UHPLC

UHPLC analyses were carried out using an UltiMate 3000 system (Thermo Scientific, Dionex Softron GmbH, Germany) with an C_18_ column (Xselect CSH 100 mm × 2.1 mm with 1.7 µm particle size; Waters Corporation, Milford, MA, USA), and the lipids samples were delivered at a flow rate of 0.25 mL/min. The injection volume was 5 µL, and the column temperature was 45 °C. Mobile phases used were acetonitrile/water (6:4 *v*/*v*) containing 10 mM ammonium formate (mobile phase A) and acetonitrile/isopropanol (1:9 *v*/*v*, mobile phase B). Lipids were separated using a gradient elution as follows [39,40]: 0–1.5 min 37% B; 1.5–4 min 37–45% B; 4–5 min 45–52% B; 5–8 min 52–58% B; 8–11 min 58–66% B; 11–14 min 66–70% B; 14–18 min 70–75% B; 18–20 min 75–98% B.

### 2.3. Lipids Quantification by Mass Spectrometric Analysis

MS was performed on a Q-Exactive plus MS (Thermo Scientific, Dionex Softron GmbH, Germany) with electrospray ionization (ESI) with heated ESI source in positive and negative mode. Nitrogen was used as both sheath gas and auxiliary gas and was set to 35 and 10 arbitrary units, respectively. The spray voltage was set to 3.2 kV for positive mode and 2.8 kV for negative mode, and ion transfer capillary was 320 °C. Higher-energy collision dissociation (HCD) with nitrogen gas and step collision energy (NCE) of 15, 25, and 35 were used to present a broader range of fragment ions. MS data were obtained in the scan range of *m*/z 240–2000 for positive mode and 200–2000 for negative mode and were processed using X calibur software version 2.2.

### 2.4. External Calibration Method

Changes in instrument sensitivity caused by degradation of lipid extracts, ion source contamination, or retention time shifts could be observed over time; therefore, the addition of quality control (QC) samples should be required to correct signal strength, retention time, or MS accuracy drifts over time [41,42]. Briefly, a pooled sample (referred to as QC) of the reconstituted extracts was prepared by combining 25 μL from each study sample. This sample was initially injected 7 times before the beginning of the run in order to condition the column. Then, the sample was re-injected once at the beginning, after every 7 injections of samples, and at the end of the run.

### 2.5. Lipidomic Data Processing

Lipid Search software 4.1.30 (Thermo Scientific, San Jose, CA, USA) was used to identify lipid molecular species and assess extractability evaluation by comparing peak abundances. The raw data were transformed into a multivariate matrix containing aligned peak areas with matched mass-to-charge ratios (*m*/*z*) and retention times and analyzed by SIMCA-P 14.1 software (Umetrics, Umea, Sweden). The differences in lipidomic signatures obtained from different protocols were examined using unsupervised principal component analysis (PCA).

## 3. Results and Discussion

### 3.1. Changes in Lipids Content

Lipid Search 4.1.30 was employed in order to process ultra-high-pressure liquid chromatography/electrospray ionization tandem mass spectrometry (UHPLC-ESI-MS/MS) records. Lipidomic analysis detected a total of 27 lipid classes and 606 compositional lipid species in *S. putrefaciens* cultivated at 30, 20, 10, 4, and 0 °C, including 17 phospholipids: cardiolipin (CA), dimethylphosphatidylethanolamine (dMePE), lysophosphatidic acid (LPA), lysophosphatidylcholine (LPC), lysophosphatidylethanolamine (LPE), lysophosphatidylglycerol (LPG), lysophosphatidylinositol (LPI), phosphatidic acid (PA), platelet-activating factor (PAF), phosphatidylcholine (PC), phosphatidylethanolamine (PE), phosphatidylethanol (PEt), phosphatidylglycerol (PG), phosphatidylinositol (PI), phosphatidylinositol (PIP), phosphatidylmethanol (PMe), and phosphatidylserine (PS); 2 glycerolipids: diglyceride (DG), and triglyceride (TG); 5 sphingolipids: ceramides (Cer), diglycosylceramide (CerG2), triglycosylceramide (CerG3), ceramide phosphate (CerP), and sphingomyelin (SM); 4 saccharolipids: digalactosyldiacylglycerol (DGDG), monogalactosyldiacylglycerol (MGDG), monogalactosylmonoacylglycerol (MGMG), and sulfoquinovosyldiacylglycerol (SQDG).

**Figure 1:**
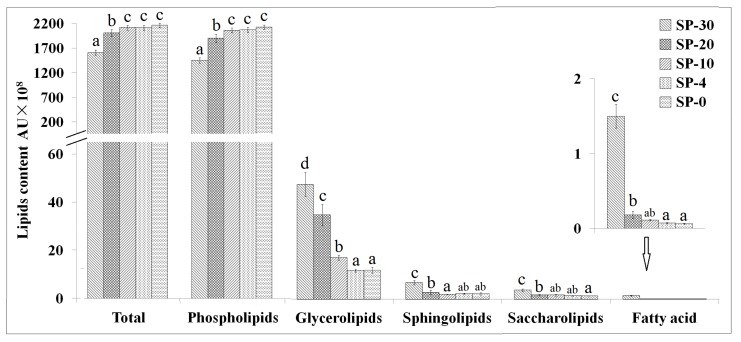
The contents of total lipids, phospholipids, glycolipids, sphingolipids, saccharolipids, and fatty acids in *Shewanella putrefaciens* cultivated at 30, 20, 10, 4, and 0 °C (*n* = 7). SP-30, *S. putrefaciens* cultivated at 30 °C; SP-20, *S. putrefaciens* cultivated at 20 °C; SP-10, *S. putrefaciens* cultivated at 10 °C; SP-4, *S. putrefaciens* cultivated at 4 °C; SP-0, *S. putrefaciens* cultivated at 0 °C. Letters above bars indicate significant differences at the *p* ≤ 0.05 level and the error bars are STDEV.

The content of total lipids (phospholipids, glycerolipids, sphingolipids, and saccharolipids) increased by 11.21% due to the cold stress at 0 °C (SP-0) compared with that of cultivated at 30 °C (SP-30, Figure 1). The contents of total lipids and phospholipids increased and the contents of glycerolipids, sphingolipids, and saccharolipids decreased with the temperature decrease for *S. putrefaciens*. Therefore, under the optimal temperature, SP-30 had significantly higher content of glycerolipids, sphingolipids, and saccharolipids compared with SP-0; however, the lower content of phospholipids (13.97%) was also found in SP-30. When *S. putrefaciens* was cultivated at 10, 4, and 0 °C, no significant differences (*p* > 0.05) in the content of total lipids and phospholipids were found among these three treatments.

The importance of lipids composition in membranes for bacteria to survive under cold stress has been generally agreed [4,33,43]. Changes in lipids response to cold stress have been reported in different species of bacteria [2,44]; however, limited information is available on lipidomics, as stated in the Introduction. In this research, the results of the lipidomic analysis suggested that *S. putrefaciens* cultivated at a lower temperature might involve some adjustments in the lipid structure of the cell membrane as phospholipids changes are important for bacteria from cold environments [37,45]. The physiological function of bacterial phospholipids is pleiotropic, which could determine the integrity and function of cells [3,46]. The elimination or a significant alteration of a specific phospholipid level can result in significant changes in cell physiology or serious damage to cell integrity [45]. In the present study, compared with SP-30, *S. putrefaciens* cultivated at lower temperature had significantly higher content of phospholipids and lower content of glycerolipids, sphingolipids, and saccharolipids.

**Figure 2:**
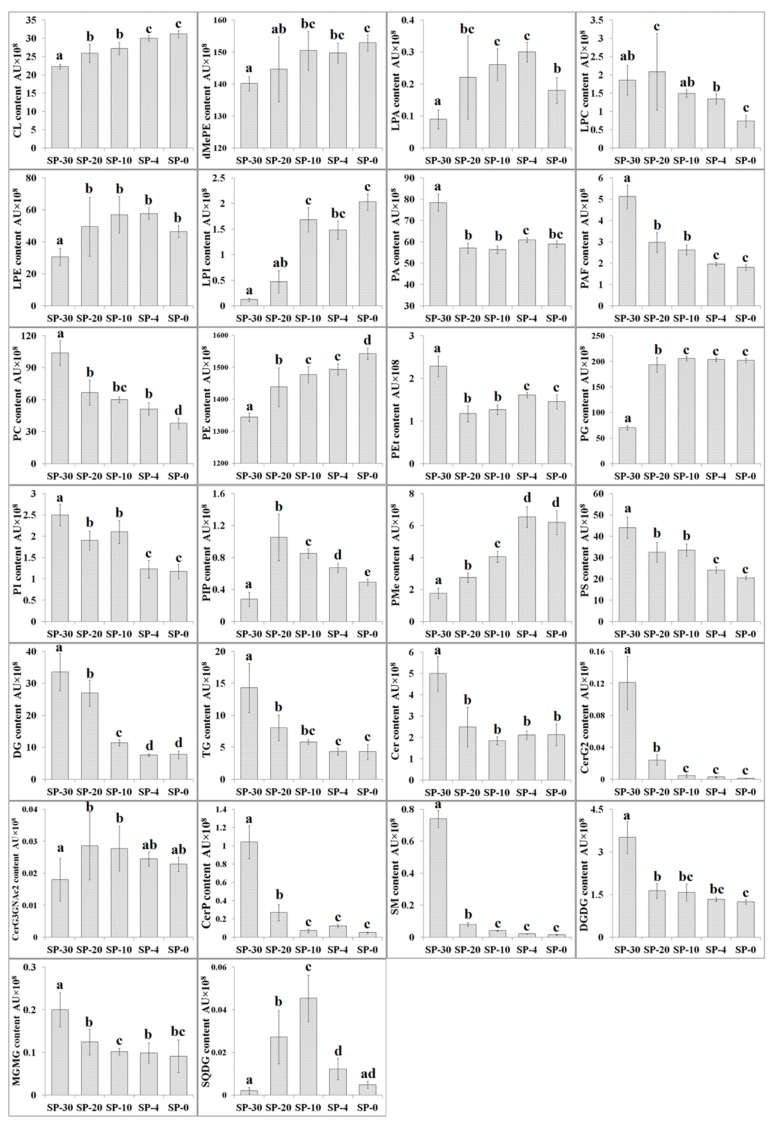
The contents of different compositional lipid classes in *S. putrefaciens* cultivated at 30, 20, 10, 4, and 0 °C (*n* = 7). SP-30, *S. putrefaciens* cultivated at 30 °C; SP-20, *S. putrefaciens* cultivated at 20 °C; SP-10, *S. putrefaciens* cultivated at 10 °C; SP-4, *S. putrefaciens* cultivated at 4 °C; SP-0, *S. putrefaciens* cultivated at 0 °C. CL, cardiolipin; dMePE, dimethylphosphatidylethanolamine; LPA, lysophosphatidic acid; LPC, lysophosphatidylcholine; LPE, lysophosphatidylethanolamine; LPG, lysophosphatidylglycerol; LPI, lysophosphatidylinositol; PA, phosphatidic acid; PAF, platelet-activating factor; PC, phosphatidylcholine; PE, phosphatidylethanolamine; PEt, phosphatidylethanol; PG, phosphatidylglycerol; PI, phosphatidylinositol; PIP, phosphatidylinositol; PMe, phosphatidylmethanol; PS, phosphatidylserine; DG, diglyceride; TG, triglyceride; Cer, ceramides; CerG2, diglycosylceramide; CerG3, triglycosylceramide; CerP, ceramide phosphate; SM, sphingomyelin; DGDG, digalactosyldiacylglycerol; MGDG, monogalactosyldiacylglycerol; MGMG, monogalactosylmonoacylglycerol; SQDG, sulfoquinovosyldiacylglycerol. Letters above bars indicate significant differences at the *p* ≤ 0.05 level and the error bars are STDEV.

The content of CL, dMePE, PE, and PG increased with the decreased temperature, whereas PAF, PC, DG, TG, CerG2, CerP, SM, and DGDG decreased under cold stress (Figure 2). *S. putrefaciens* cultivated at 0 °C had a significant increase (*p* < 0.05) in the content of CL, dMePE, LPA, LPE, LPI, PE, PG, PIP, PMe, and MGMG, whereas declined contents of LPC, PA, PAF, PC, PEt, PI, PS, DG, TG, Cer, CerG2, CerP, SM, and DGDG were observed. Alterations in cultivated temperature also induced significant changes in the ratio of MGDG to DGDG [47]. *S. putrefaciens* cultivated at lower temperature resulted in a higher MGDG/DGDG ratio, which was an adaptive response to increasing membrane disorder. This change is considered as a compensatory mechanism to keep the biophysical properties of the cell membrane close to the lamellar to hexagonal phase transition. MGDG forms inverted non-lamellar structures as opposed to the bilayer conformation of DGDG. By introducing non-bilayer lipids into the cell membrane, the bacterial cell membrane is kept at a stable limit to respond flexibly to extracellular stimuli that interfere with the biophysical properties [48].

However, the mechanism is not clear, and the activities of interfacial glycosyltransferases may be regulated by the physical properties of substrates containing glycosyltransferases [49]. The higher MGDG/DGDG ratio in *S. putrefaciens* cultivated at lower temperature could contribute to maintain the cell membrane fluidity to enhance the cold adaption.

Phospholipids are the major components of bacterial cell membrane structures, and some species have also been recognized as signaling molecules affecting bacterial stress responses by activating specific protein phosphatases and kinases, mediating reactive oxygen generation, and altering cytoskeletal networks [45,50,51,52]. The dominating phospholipids were PE, followed by PG and PC (Figure 2). The relationship of PG and cold stress has been well investigated [53,54]. In the present research, the content of PG and PE were increased to be the main phospholipids component of the membrane in *S. putrefaciens* cultivated at lower temperature, which suggested that the increased content of PG could help maintain the stability of bacterial cell membranes under cold stress. However, the content of PA and PC decreased in response to cold stress for *S. putrefaciens*. PA is recognized to be an important lipid second messenger, regulating lipid metabolism and cytoskeleton dynamics, and affecting other signaling pathways [55,56,57,58]. Redón et al. [59] studied the effect of growth temperature (13 and 30 °C) on lipid composition of different *Saccharomyces* species showing that the content of triacylglyceride and medium-chain fatty acids increased when cultivated at low temperature, whereas the content of PA and the PC/PE ratio decreased. Klose et al. also showed PE increasing and that of PI is inconsistent with decreasing temperature [60]. The PI content of SP-30 was higher than those cultivated at lower temperature, and this trend was contrary to PE, which tried to explain the structural changes of polar head groups [61]. The volume of the relevant phospholipid molecules in bacterial cell membranes decreased with the lower temperature, and molecular volumes for lipids composed of palmitoyl-oleoyl chains and different head groups varied with temperature. Similar research was done by Torija et al. [62] who showed that lipids composition changed with the growth temperature, and the optimal fluidity of the membrane at low temperatures was regulated by changes of unsaturation degree. PC is an important structural and functional phospholipid in bacterial cell membranes and plays an important role in signal transduction as it is a major source of lipid secondary messengers, such as PA, LPC, LPA, and diacylglycerol [63]. The decreased contents of PC were also observed in *S. putrefaciens* cultivated at lower temperature, which coincides with the low abundance of PC detected in *Bacillus subtilis* under cold stress [64].

Lysophospholipids are generated as metabolic intermediates in phospholipid synthesis or from bacterial membrane degradation [65]. Besides playing an important role in phospholipid metabolism, lysophospholipid also has the function of second messenger and has extensive biological activities [66]. Lysophospholipids made up about 1.72% in SP-30 and increased to 2.32% in SP-0 and are mostly found in the form of LPE. Although the content of lysophospholipids is small, they are essential components of bacterial cell membranes. The change of lysophospholipid content could affect the spontaneous curvature of cell membrane and the conformation of ion channel [67]. LPE contents increased in *S. putrefaciens* cultivated at lower temperature, coinciding with the study results of Nina et al. that the environmental stress led to a significant increase in the content of LPE in *Yersinia pseudotuberculosis* [68]. The increased content of LPE led to an increase in the phase transition temperature of the cell membrane with a greater membrane rigidity [68,69].

### 3.2. Changes in Content of Different Compositional Lipid Species

**Figure 3:**
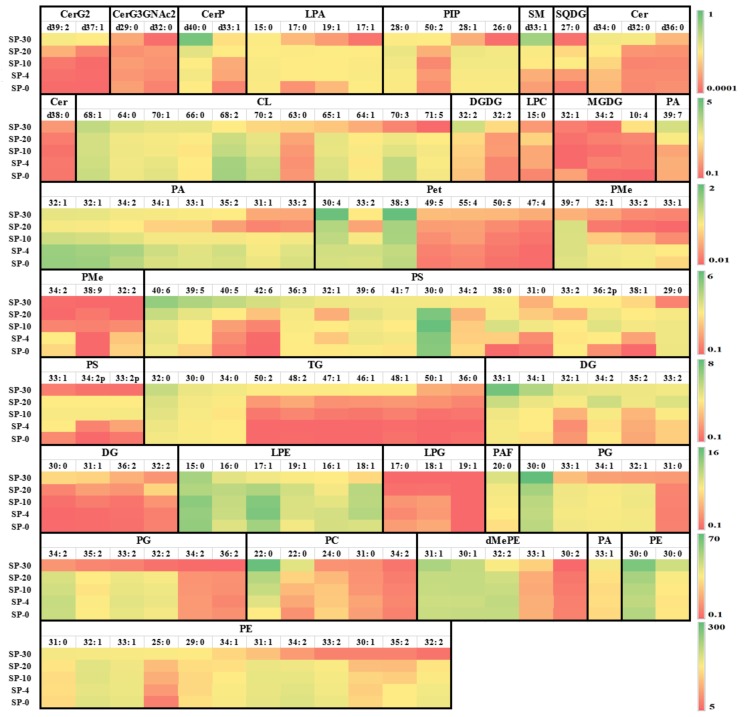
The contents of some main compositional lipid molecules (AU × 10^8^) in *S. putrefaciens* cultivated at 30, 20, 10, 4, and 0 °C (*n* = 7). SP-30, *S. putrefaciens* cultivated at 30 °C; SP-20, *S. putrefaciens* cultivated at 20 °C; SP-10, *S. putrefaciens* cultivated at 10 °C; SP-4, *S. putrefaciens* cultivated at 4 °C; SP-0, *S. putrefaciens* cultivated at 0 °C. CA, cardiolipin; dMePE, dimethylphosphatidylethanolamine; LPA, lysophosphatidic acid; LPC, lysophosphatidylcholine; LPE, lysophosphatidylethanolamine; LPG, lysophosphatidylglycerol; LPI, lysophosphatidylinositol; PA, phosphatidic acid; PAF, platelet-activating factor; PC, phosphatidylcholine; PE, phosphatidylethanolamine; PEt, phosphatidylethanol; PG, phosphatidylglycerol; PI, phosphatidylinositol; PIP, phosphatidylinositol; PMe, phosphatidylmethanol; PS, phosphatidylserine; DG, diglyceride; TG, triglyceride; Cer, ceramides; CerG2, diglycosylceramide; CerG3, triglycosylceramide; CerP, ceramide phosphate; SM, sphingomyelin; DGDG, digalactosyldiacylglycerol; MGDG, monogalactosyldiacylglycerol; MGMG, monogalactosylmonoacylglycerol; SQDG, sulfoquinovosyldiacylglycerol.

The ESI-MS/MS analysis identified PE (30:0), PE (15:0/15:0), PE (31:0), PA (33:1), PE (32:1), PE (33:1), PE (25:0), PC (22:0), PE (29:0), PE (34:1), dMePE (15:0/16:1), PE (31:1), dMePE (15:1/15:0), PG (34:2), and PC (11:0/11:0) as the most abundant compositional lipid species among the 606 lipids detected in *S. putrefaciens* cultivated at 30, 20, 10, 4, and 0 °C. Figure 3 illustrates the change of some higher content compositional lipid species. *S. putrefaciens* cultivated at lower temperature significantly resulted in significant accumulation of compositional lipid species containing long and very long chain saturated fatty acyls, e.g., CL (68:2, 70:2, 70:3) and PS (29:0, 30:0). The increase in long- and very long fatty acids’ levels may be due to the increased activity of fatty acid elongase [70]. Lipidomic analysis also found several specific compositional lipid species in *S. putrefaciens* responding to enhance cold tolerance. For example, SP-20 had the lowest contents of PA molecules (32:1, 34:2, 34:1, 33:1, 35:2, 31:1, and 33:2). It is interesting to identify unsaturated bioactive LPC and LPE from the perspective of lipid metabolism and membrane structure, composition, and dynamics of these psychrophiles, which may regulate the activities of regulatory and signaling proteins [71].

### 3.3. Changes in the Unsaturation Level of Compositional Lipid Species in Response to Cold Stress

**Figure 4:**
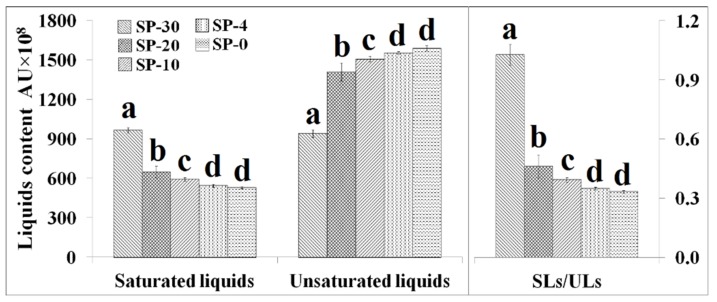
The contents of saturated liquids (AU × 10^8^), unsaturated liquids (AU × 10^8^), and the ratio of saturated/unsaturated liquids (SLs/ULs) in *S. putrefaciens* cultivated at 30, 20, 10, 4, and 0 °C (*n* = 7). SP-30, *S. putrefaciens* cultivated at 30 °C; SP-20, *S. putrefaciens* cultivated at 20 °C; SP-10, *S. putrefaciens* cultivated at 10 °C; SP-4, *S. putrefaciens* cultivated at 4 °C; SP-0, *S. putrefaciens* cultivated at 0 °C. Letters above bars indicate significant differences at the *p* ≤ 0.05 level and the error bars are STDEV.

As shown in Figure 4, *S. putrefaciens* cultivated at low temperature significantly increased the total unsaturated compositional liquids content but decreased the content of saturated compositional liquids content. We employed the ratio of saturated/unsaturated compositional liquids (SLs/ULs) to indicate the degree of unsaturation of membrane lipids; a high ratio of SLs/ULs indicates the presence of more highly saturated membrane lipids, and vice versa. From SP-30 to SP-20, the changes in the ratios of SLs/ULs were big and the ratios of SLs/ULs were found to be lower at lower temperature. Bacterial cell membranes become more rigid at low temperatures, and chemical changes occur in some membrane fatty acids to prevent cellular damage [36,72]. The maintenance of bacterial membrane fluidity plays an important role in various physiological functions of cells, such as the transport of nutrients, the protection of adverse environment, and cell morphology [73]. The membrane lipid bilayers undergo a reversible change of state from a fluid (disordered) to a non-fluid (ordered) array of the fatty acyl chains. Phospholipids that contain UFAs have much lower transition temperatures than those lipids made of SFAs. SFA acyl chains can pack tightly, but the steric hindrance imparted by the rigid kink of the cis double bond results in much poorer chain packing of UFAs, even below the phase transition temperature. Therefore, lower temperatures result in an increase in the number of UFAs in the membrane [74,75]. The presence of cis double bonds in the membrane lipid acyl chains could be interfered with the acyl chain packing, resulting in poorer packing of the acyl chains and lower gel–liquid crystal phase transition temperature of the membrane. Therefore, unsaturated lipids are key molecules that regulate the fluidity of cell membranes [76].

## 4. Conclusions

Lipidomic analysis suggested that the changes of lipid metabolism in *S. putrefaciens* could be better adapted to low-temperature environment as manifested by (a) the increase of PG content that contributes to the construction of membrane lipid bilayer and successfully maintains membrane integrity and normal protein function under low-temperature stress; (b) the increased content of unsaturation levels for lipids; and (c) reducing lipid signaling or second messenger molecules and their precursors, such as PA, PC, and PI, thereby inactivating downstream pathways and protecting *S. putrefaciens* from cold damages. The underlying biochemical and molecular mechanisms of specific compositional lipid species in response to cold stress are not well understood and deserve further investigation. Precise quantification of specific compositional lipid species by TLC and GC coupled with flame ionization detector may provide additional information on how individual lipids participate in cold stress tolerance.
